# Mechanical characterization and force-displacement hysteretic curves from in-plane cyclic tests on strong masonry infills

**DOI:** 10.1016/j.dib.2017.12.015

**Published:** 2017-12-14

**Authors:** Paolo Morandi, Sanja Hak, Guido Magenes

**Affiliations:** a University of Pavia and EUCENTRE, Via Ferrata 1, 27100 Pavia, Italy; b University of Zagreb, Croatia and University of Pavia, Italy

## Abstract

This article contains information related to a recent study “Performance-based interpretation of in-plane cyclic tests on RC frames with strong masonry infills” (Morandi et al., 2017 [1]). Motivated by the necessity to improve the knowledge of the in-plane seismic response of rigid strong masonry infills, a wide experimental campaign based on in-plane cyclic tests on full-scale RC infilled frame specimens, supplemented with a complete characterization of the materials, has been conducted at the laboratory of the Department of Civil Engineering and Architecture of the University of Pavia. The masonry is constituted by vertically perforated 35 cm thick clay units with tongue and groove and dry head-joints and general-purpose mortar bed-joints. The paper reports the results of the mechanical characterization and of the force-displacement hysteretic curves from the in-plane cyclic tests.

**Specifications Table**

**Table:** 

Subject area	*Engineering*
More specific subject area	*Earthquake engineering*
Type of data	*Tables, images, text file, graphs, figures*
How data was acquired	*Acquisition from the controller of the actuator, the linear potentiometers and optical markers*
Data format	*Raw and analysed data*
Experimental factors	*–*
Experimental features	*Tests of material characterization and in-plane cyclic tests*
Data source location	*–*
Data accessibility	*Data provided in the article is accessible to the public*

**Value of the data**

• Data in this work may improve the understanding of in-plane cyclic response of strong masonry infills
• Lack of data regarding in-plane experimental tests on strong masonry infills
• Full scale specimens
• Complete characterization of the materials (concrete and reinforcing steel, clay units, mortar and masonry)

## 1 Data

The data refer to the results of the mechanical characterization and of the in-plane cyclic tests on a strong masonry infill typology. The tests have been conducted at the laboratory of the Department of Civil Engineering and Architecture of the University of Pavia.

The description of the infill typology is reported in Morandi et al. [1].

### 1.1 Material characterization

A complete characterization of the relevant properties for all materials utilized for the construction of the specimens has been carried out. Principally, the evaluation of clay units, mortar and masonry properties for the selected infill typology was of primary importance. The results of the tests of characterization are described and reported in detail in Section 2.1.

### 1.2 In-plane cyclic tests

A series of cyclic pseudo-static in-plane tests has been carried out on bare and fully or partially infilled full-scale single-storey, single-bay RC frames, designed according to European code provisions. In particular, one RC frame was tested without infill (TNT) up to maximum drift of 3.50% in order to reach the ultimate conditions of the specimen, while three fully infilled specimens (TA1, TA2 and TA3) were tested at three increasing maximum levels of drift, equal to 1.00, 1.50 and 2.50%. In addition, a partially infilled frame configuration with a 1.44 m wide and full-height opening in the middle of the span (TA4) was tested, reaching a maximum in-plane drift of 1.00%. The in-plane infill performance at increasing levels of drift was aimed to approximately represent different limit state conditions. The type and the dimensions of the tested frames are reported in Morandi et al. [1].

## 2 Results of the experimental campaign

### 2.1 Material characterisation

A detailed characterisation of the relevant properties for all materials utilised for the construction of the specimens has been carried out and presented in the following subsections.

#### 2.1.1 Concrete and steel reinforcement properties

The characterization on the materials of the RC frames has included compression tests on 6 concrete cubes (of 150×150×150 mm dimensions) for columns and 6 for beams, according to European standard EN 12390-3:2009 [2], and tension tests on reinforcement rebars, following European standards EN ISO 15630-1:2010 [3] and EN ISO 6892-1:2016 [4]; the specimens have been sampled during the construction phase. The related results are reported in Table 1.

**Table 1:** Summary of strength results on concrete cubes and steel rebars.

**Property**	**Symbol**	**Mean** (Mpa)	**c.o.v.** (%)
Compr. strength on cubes of concrete C28/35 (columns)	*R*_*c,col*_	34.0	3.6
Compr. strength on cubes of concrete C28/35 (beams)	*R*_*c,beam*_	34.3	3.1
Yield. tensile strength of steel rebars B450C (Φ8)	*f*_*y*_	530	1.7
Ult. tensile strength of steel rebars B450C (Φ8)	*f*_*u*_	600	1.5
Yield. tensile strength of steel rebars B450C (Φ10)	*f*_*y*_	529	1.4
Ult. tensile strength of steel rebars B450C (Φ10)	*f*_*u*_	616	0.2
Yield. tensile strength of steel rebars B450C (Φ14)	*f*_*y*_	538	2.8
Ult. tensile strength of steel rebars B450C (Φ14)	*f*_*u*_	608	2.9
Yield. tensile strength of steel rebars B450C (Φ22)	*f*_*y*_	491	0.3
Ult. tensile strength of steel rebars B450C (Φ22)	*f*_*u*_	601	0.3

#### 2.1.2 Masonry unit and mortar properties

A summary of characterisation tests on masonry units and mortar is given in Table 2.

**Table 2:** Summary of characterisation tests on masonry units and mortar.

**Type of specimen**	**Type of test**	**Number of specimens**
Masonry units	Vertical compression strength	30
	Horizontal compression strength	10
Mortar prisms	Flexural tension strength	3
	Compression strength	3

The measurement of the compression strength of the units was carried out following European norms EN 771-1:2011+A1:2015 [5] and EN 772-1:2011+A1:2015 [6]. With the aim to evaluate, in addition to the average vertical compression strength of the masonry units, also the characteristic value, as indicated in the explanatory of the Italian National Code (NTC08 *Circolare esplictiva* [7]), 30 units have been tested in vertical compression. A summary of the results obtained from tests of vertical and horizontal compression strength is given in Table 3.

**Table 3:** Summary of compression test results on masonry units.

	**Vertical compression**		**Horizontal compression**	
	***f*** [MPa]	***f***_***norm***._ [MPa]	***f*** [MPa]	***f***_***norm***._ [MPa]
Mean strength, *f*_*b*_	8.64	9.81	2.78	3.15
St. dev.	0.79	–	0.30	–
C.o.v.	9.2%	–	10.8%	–
Characteristic strength, *f*_*bk*_	7.34	–	1.95	–

According to the norm related to mortar testing (EN 1015-11:1999+A1:2006 [8]), flexural and compressive tests on three prism-shaped specimens of mortar (of 40×40×160 mm dimensions) were carried out for each batch of mortar. Average values of flexural and compressive strength equal to 2.15 MPa and 7.68 MPa, respectively, have been found.

#### 2.1.3 Masonry properties

For the characterisation tests on wallettes, related to compression strength, well established procedures and test setups, applied in previous experimental campaigns have been implemented. The test setup for the evaluation of the initial shear strength on masonry triplets from a series of shear tests under increasing levels of compression has been recently developed (see Morandi et al. [9]). A summary of characterisation tests on masonry specimens is given in Table 4.

**Table 4:** Summary of characterisation tests on masonry.

**Type of specimen**	**Type of test**	**No. of specimens**
Masonry wallettes	Vertical compression strength	6
	Horizontal compression strength	6
Masonry triplets	Initial shear strength	9

In relation with the resistance of masonry as a composite material, the requirements related to the determination of the vertical and the horizontal strength on masonry wallettes are defined in EN 1052-1:1998 [10]. For both directions, six specimens have been tested. On all masonry units and wallettes to be tested, in the regions of support or force application, a cement mortar topping of about 1.0 *cm* was applied with the aim to ensure even contact surfaces.

##### 2.1.3.1 Vertical compression strength

**Fig. 1:**
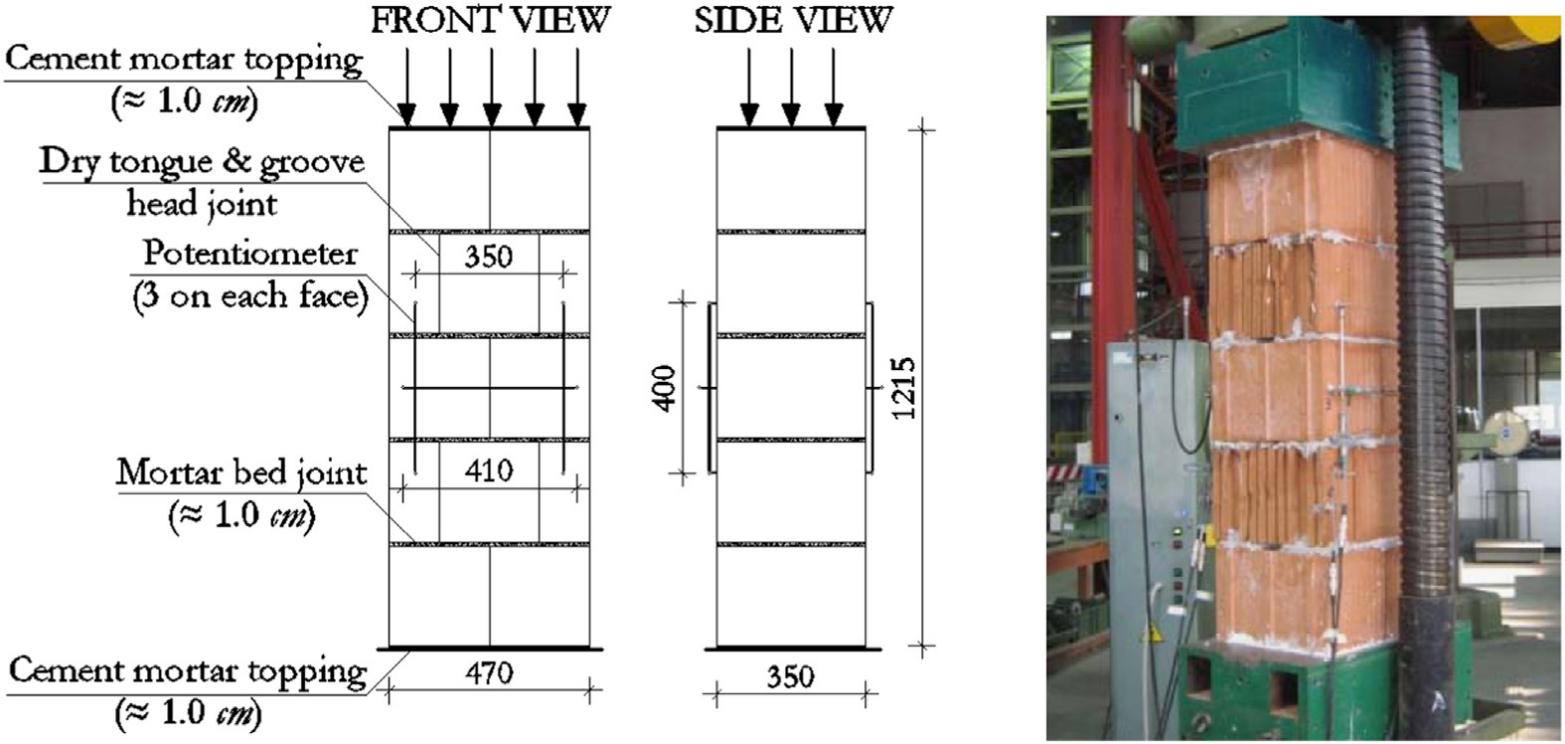
Masonry characterisation – details and instrumented specimen: Vertical compression.

**Fig. 2:**
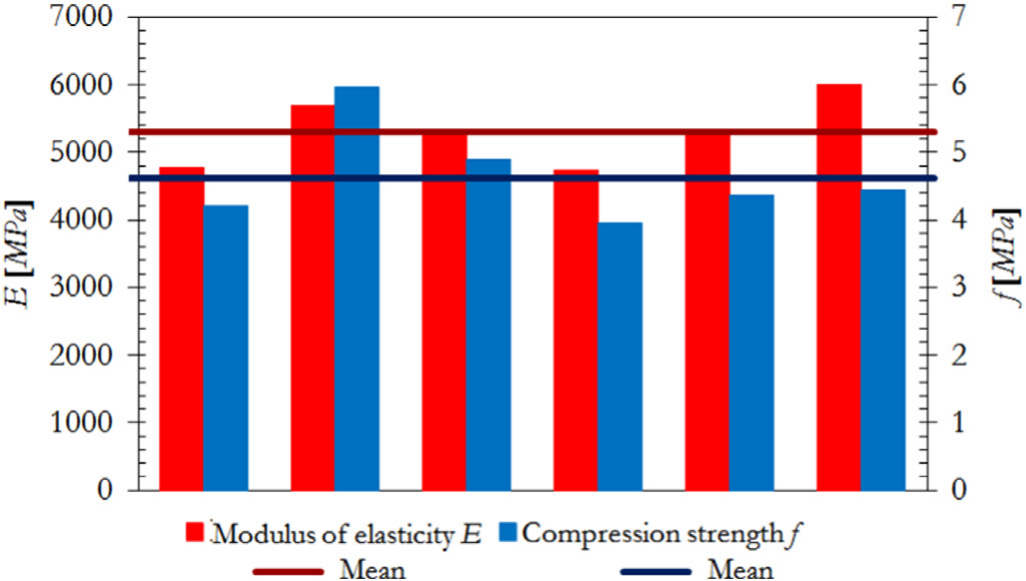
Masonry characterisation test results: vertical compression.

**Fig. 3:**
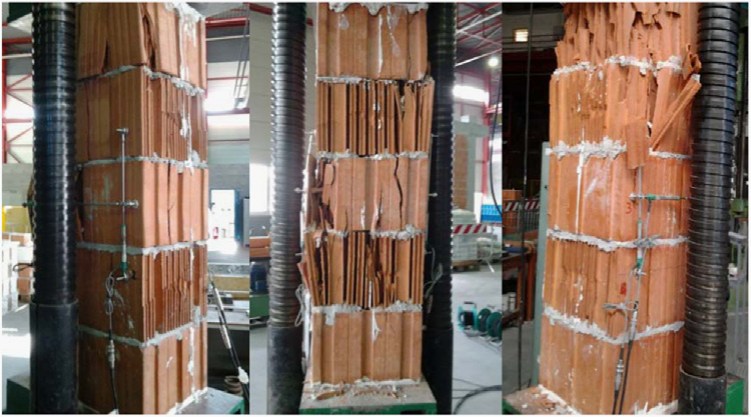
Damaged masonry wallettes after vertical compression tests.

The dimensions, instrumentation and construction of the wallettes for vertical compression tests are illustrated in Fig. 1, while the corresponding results are summarized in Table 5 and Fig. 2. Examples of typical failure patterns obtained due to vertical compression are shown in Fig. 3.

**Table 5:** Summary of vertical compression test results on masonry wallettes.

**Specimen no.**	***f***_***vert***_ [MPa]	***E***_***vert***_ [MPa]		***f***_***vert***_ [MPa]	***E***_***vert***_ [MPa]
1	4.21	4780	**Mean strength*****f***_***vert,m***_	**4.64**	**5299**
2	5.96	5686			
3	4.89	5278	**St. dev.**	0.66	455
4	3.96	4735	**C.o.v.**	14.1%	8.6%
5	4.36	5311	**Characteristic strength*****f***_***vert,k***_	**3.86**	–
6	4.44	6007			

##### 2.1.3.2 Lateral compression strength

**Fig. 4:**
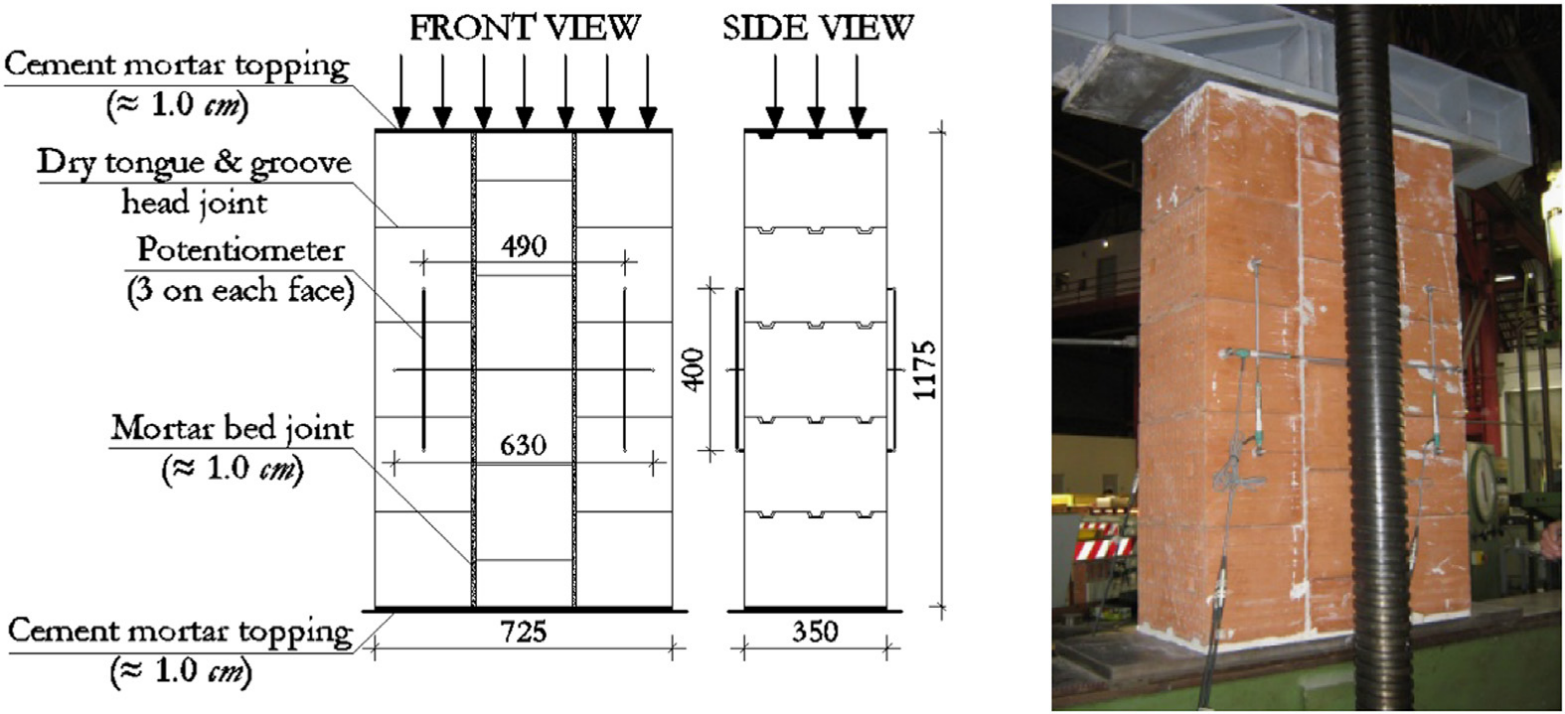
Masonry characterisation – details and instrumented specimen: Lateral compression.

**Fig. 5:**
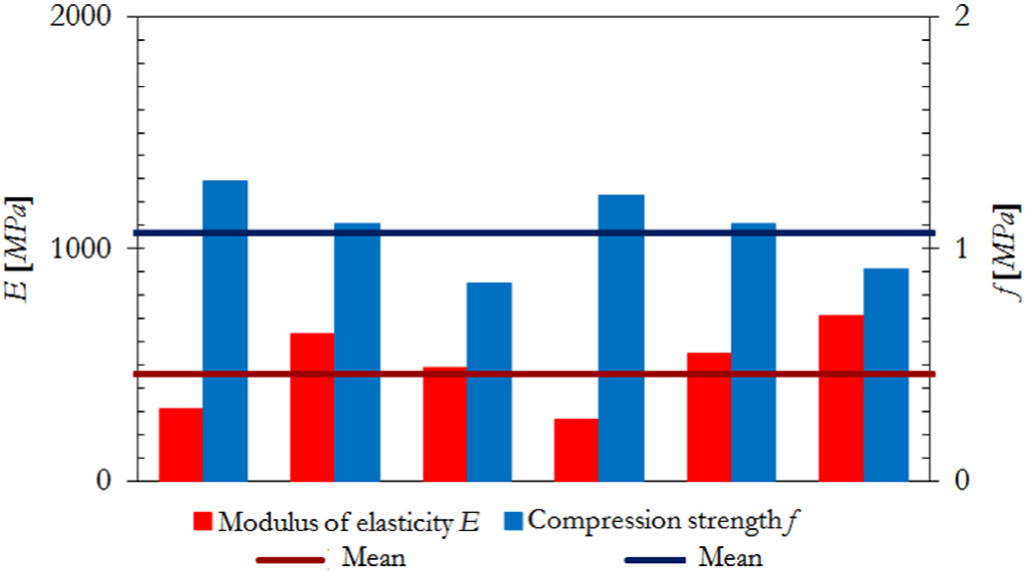
Masonry characterisation test results: Lateral compression.

**Fig. 6:**
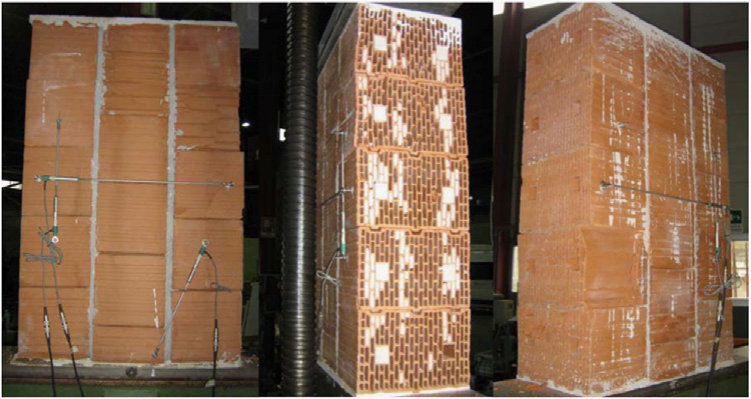
Damaged masonry wallettes after lateral compression tests.

The dimensions, instrumentation and construction of the wallettes for lateral compression tests are illustrated in Fig. 4, while the corresponding results are summarized in Table 6 and Fig. 5. Examples of typical failure patterns obtained due to lateral compression are shown in Fig. 6.

**Table 6:** Summary of lateral compression test results on masonry wallettes.

**Specimen no.**	***f***_***lat***_ [MPa]	***E***_***lat***_ [MPa]		***f***_***lat***_ [MPa]	***E***_***lat***_ [MPa]
1	1.29	310	**Mean strength**	**1.08**	**494**
2	1.11	635	***f***_***lat,m***_		
3	0.85	488	**St. dev.**	0.16	162
4	1.23	265	**C.o.v.**	14.5%	32.8%
5	1.11	550	**Characteristic strength**	**0.85**	–
6	0.91	712	***f***_***lat,k***_		

##### 2.1.3.3 Shear strength

**Fig. 7:**
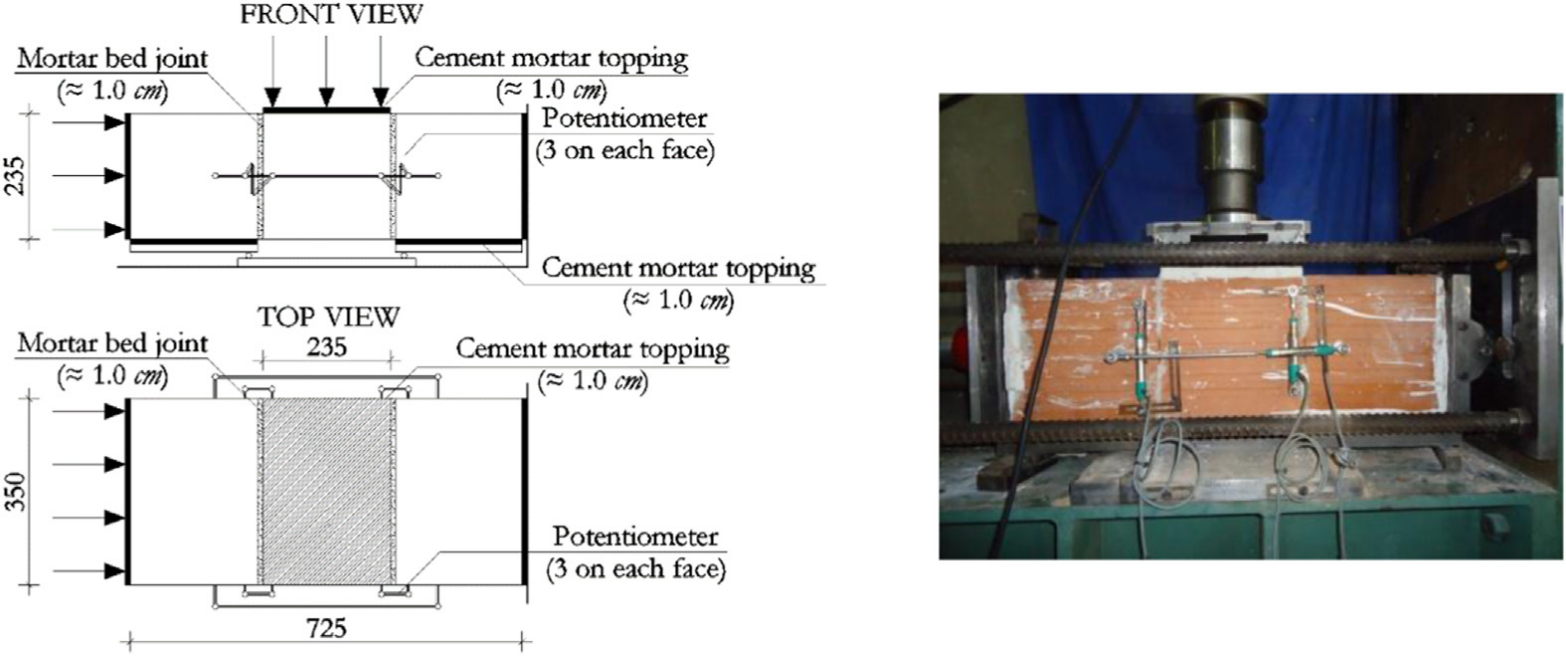
Masonry characterisation – details and instrumented specimen: shear strength.

The initial masonry shear strength in the plane of horizontal mortar bed joints has been evaluated following the requirements given in EN 1052-3:2002+A1:2007 [11], subjecting masonry triplets to a series of shear tests. Specifically, three samples have been tested in the direction parallel to the bed-joints at each of three increasing levels of compression in the direction orthogonal to the bed-joints, as illustrated in Fig. 7.

**Fig. 8:**
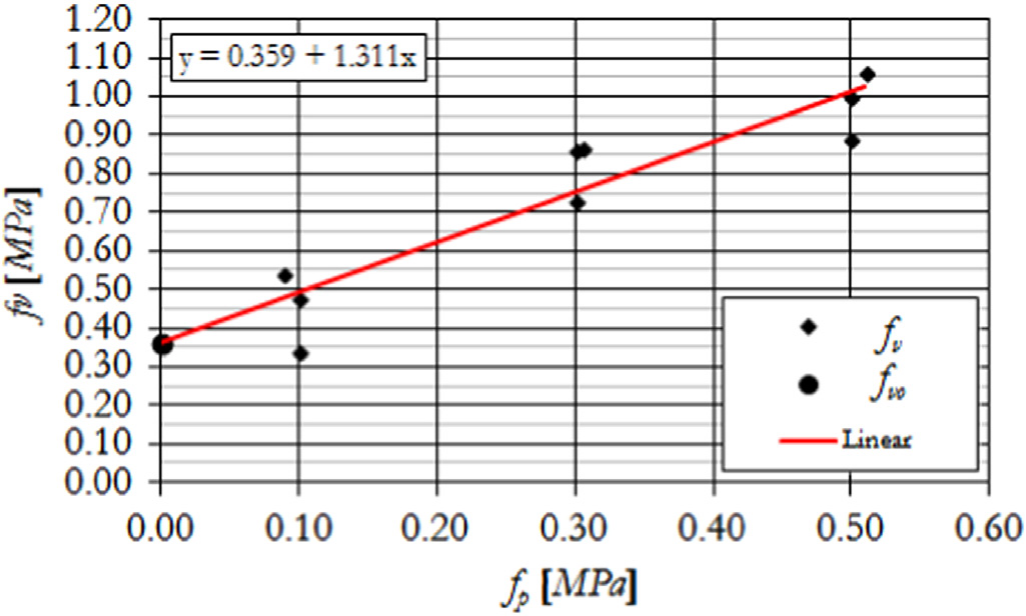
Characterisation test results: Initial shear strength.

**Fig. 9:**
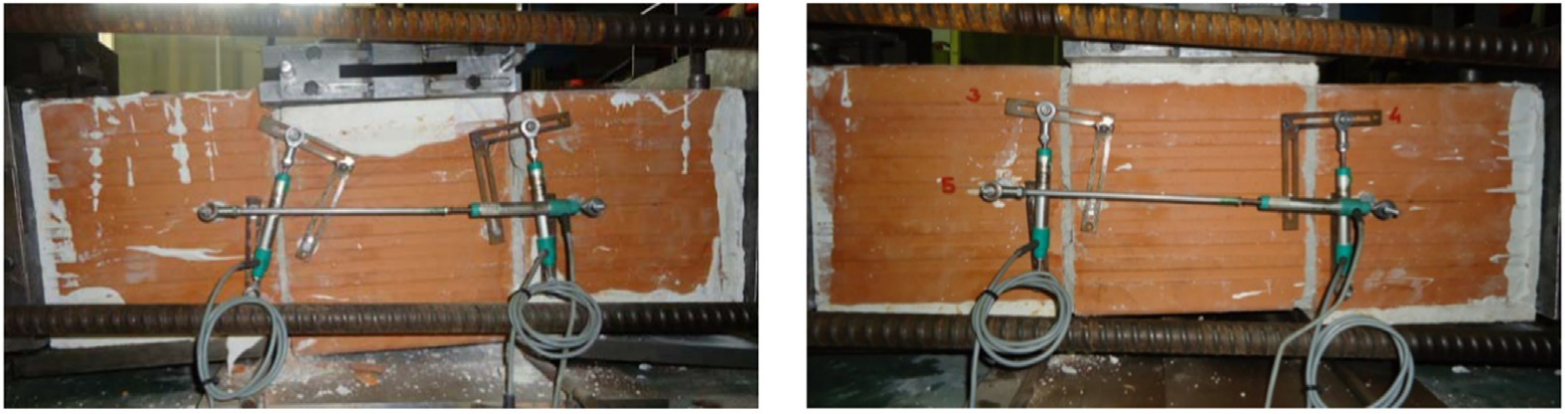
Damaged masonry triplets after shear strength tests.

The corresponding results are summarized in Table 7 and Fig. 8, illustrating the evaluation of the initial shear strength under zero compression *f*_*v0*_ and the corresponding characteristic value *f*_*v0,k*_, as well as the corresponding friction coefficient *µ*=*tanα* and its characteristic value *µ*_*k*_*=tanα*_*k*_. Examples of typical failure patterns obtained during the shear sliding tests are shown in Fig. 9.

**Table 7:** Summary of shear strength test results on masonry triplets.

**Specimen no.**	***f***_***p***_ [MPa]	***F***_***max***_ [kN]	***f***_***v***_ [MPa]		
1	0.10	55	0.34	***Mean strength***	**0.36**
2	0.30	142	0.87	***f***_***v0,m***_	
3	0.50	163	1.00	***Characteristic strength***	**0.29**
4	0.51	173	1.06	***f***_***v0,k***_	
5	0.50	145	0.89	***Mean friction coeff.***	**1.31**
6	0.30	120	0.73	***µ=tan α***	
7	0.30	141	0.86	***Charact. friction coeff.***	**1.05**
8	0.09	87	0.54	***µ***_***k***_***=tan α***_***k***_	
9	0.10	78	0.48		

### 2.2 Cyclic in-plane tests

#### 2.2.1 Testing protocol: procedure and loading history

In order to perform the in-plane tests, the specimens have been placed in position and attached to the strong floor and the actuator has been secured, allowing free rotation of the actuator hinges. The system used to introduce horizontal forces, consisting of two steel plates and four steel bars has been placed on the beam of the specimen and fixed introducing adequate precompression. Out-of-plane restraints have been brought in position to prevent possible out-of-plane displacements of the specimen.

Subsequently, using the system for vertical force introduction, on both columns simultaneously, the load has been imposed by the hydraulic jacks at a velocity of about 2.0 kN/s. The achieved vertical load of 400 kN per column has been kept constant during the entire test. Finally, reverse cycles of horizontal in-plane loading (first pull, then push) were imposed on the frame in the height of the beam by means of the servo-controlled hydraulic actuator. Firstly, two different levels of force-controlled loading were accomplished, while subsequently displacement-controlled loading cycles at increasing levels of in-plane drift were imposed, as summarised in Table 8. Clearly, not all levels of drift have been achieved in each test. For each level of loading (target force or displacement) three complete reverse loading cycles have been carried out and the duration of load application has been kept approximately constant.

**Table 8:** In-plane loading cycles.

**Target level**	**Drift** [%]	**Displacement** [mm]	**Force** [mm]	**Velocity** [mm/s]	**Velocity** [kN/s]	**Duration/cycle** [s]
**01F**	*δ*_*1*_	*δ*_*1*_*H*	≈ 0.15 *F*_*max*_	–	4×0.15 *F*_*max*_/200	200
**02F**	*δ*_*2*_	*δ*_*2*_*H*	≈ 0.30 *F*_*max*_	–	4×0.30 *F*_*max*_/200	200
**01D**	0.05	1.56	*F*_1_	0.025	–	250
**02D**	0.10	3.13	*F*_2_	0.050	–	250
**03D**	0.15	4.69	*F*_3_	0.075	–	250
**04D**	0.20	6.25	*F*_4_	0.100	–	250
**05D**	0.25	7.81	*F*_5_	0.125	–	250
**06D**	0.30	9.38	*F*_6_	0.150	–	250
**07D**	0.35	10.94	*F*_7_	0.175	–	250
**08D**	0.40	12.50	*F*_8_	0.200	–	250
**09D**	0.50	15.63	*F*_9_	0.250	–	250
**10D**	0.60	18.75	*F*_10_	0.300	–	250
**11D**	0.80	25.00	*F*_11_	0.400	–	250
**12D**	1.00	31.25	*F*_12_	0.500	–	250
**13D**	1.25	39.06	*F*_13_	0.625	–	250
**14D**	1.50	46.88	*F*_14_	0.750	–	250
**15D**	1.75	54.69	*F*_15_	0.875	–	250
**16D**	2.00	62.50	*F*_16_	1.000	–	250
**17D**	2.50	78.13	*F*_17_	1.250	–	250
**18D**	3.00	93.75	*F*_18_	1.500	–	250
**19D**	3.50	109.38	*F*_19_	1.750	–	250

#### 2.2.2 Instrumentation

**Fig. 10:**
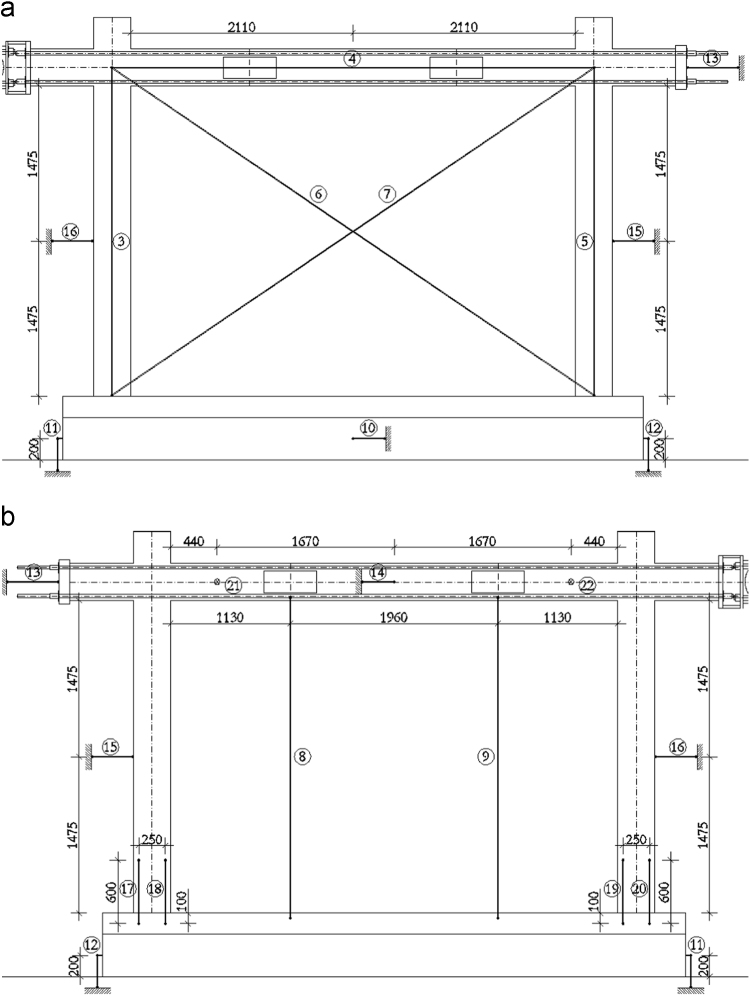
Bare frame specimen instrumentation – in-plane: (a) Front view; (b) Back view.

**Fig. 11:**
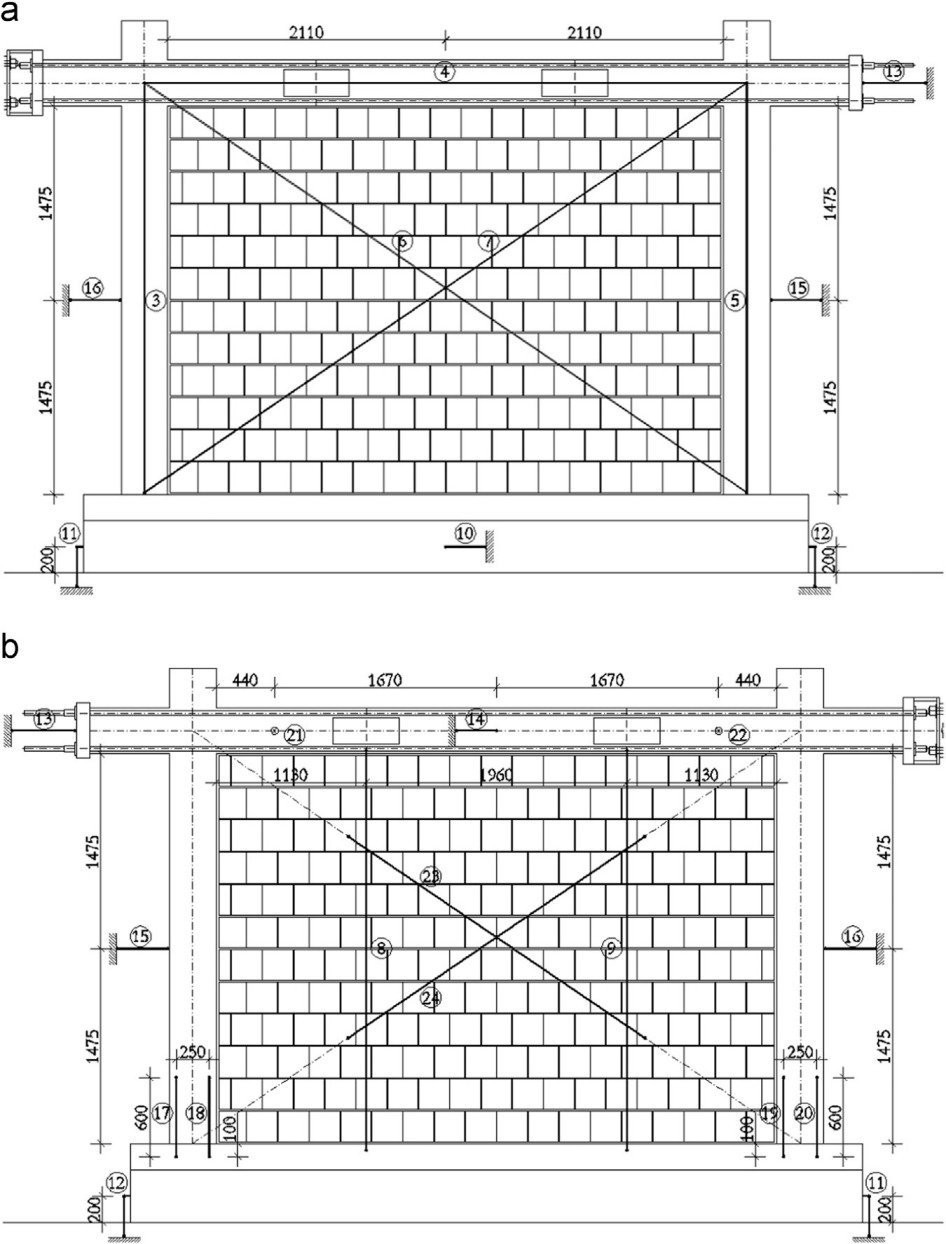
Infilled frame specimen instrumentation (TA1_IP, TA2_IP & TA3_IP): (a) Front view; (b) Back view.

**Fig. 12:**
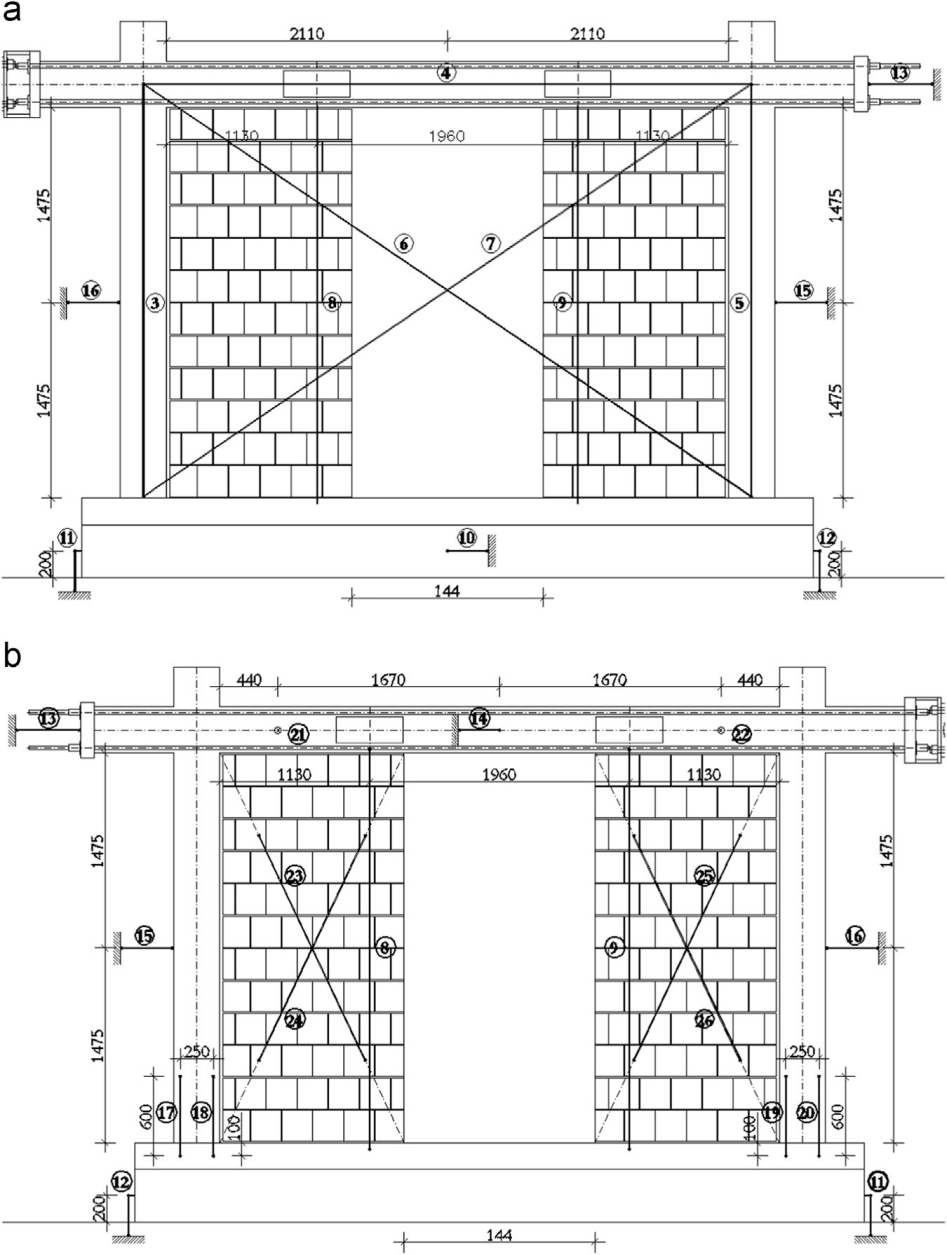
Infilled frame specimen instrumentation (TA4_IP): (a) Front view; (b) Back view.

In order to measure the displacements and deformations of the specimen during the in-plane tests, displacement transducers (linear potentiometers) have been adopted, i.e. in total, 22 potentiometers have been used for the bare frame (TNT_IP), 24 for the fully infilled frame configurations (TA1_IP, TA2_IP and TA3_IP), and 32 for the partially infilled frame (TA4_IP). The layouts of the displacement transducers adopted for the bare frame and the infilled frame specimens are schematically represented in Fig. 10, Fig. 11, Fig. 12.

The centreline deformation of the RC frame has been measured using a typical instrumentation scheme consisting of potentiometers along the column heights (3, 5), the beam length (4) and the corresponding frame diagonals (6, 7). In addition, to record the deformed shape of the beam, vertical displacements of the beam have been measured in two points within the span of the specimen (8, 9). Horizontal displacements of the frame have been measured on top at the centreline of the beam (13 at the beam end, 14 at the centre of the beam) as well as at the half-height of the column (15, 16). Deformations in the plastic hinge regions at the bottom of the columns have been recorded using pairs of potentiometers (17 and 18, 19 and 20) for each column. Any potential horizontal displacement of the foundation has been measured at the foundation centre on one side of the specimen (10), while the occurrence of possible uplift has been recorded using one potentiometer on each end of the foundation (11, 12). In order to control the out-of-plane displacements of the specimen during in-plane loading, two potentiometers (21, 22) have been placed in the beam centreline within the frame span orthogonally to the plane of the specimen. For the fully infilled frame specimens deformations of the masonry infill were measured using two potentiometers (23, 24) placed on the diagonals in the central part of the panel. In the case of partial infill five potentiometers were placed on each part of the wall, two in the vertical direction (27 and 29, 30 and 32), one at the top of the infill (28, 31) and two diagonally (23 and 24, 25 and 26). In addition, displacements possibly occurring in the actuator hinges have also been recorded (1, 2).

**Fig. 13:**
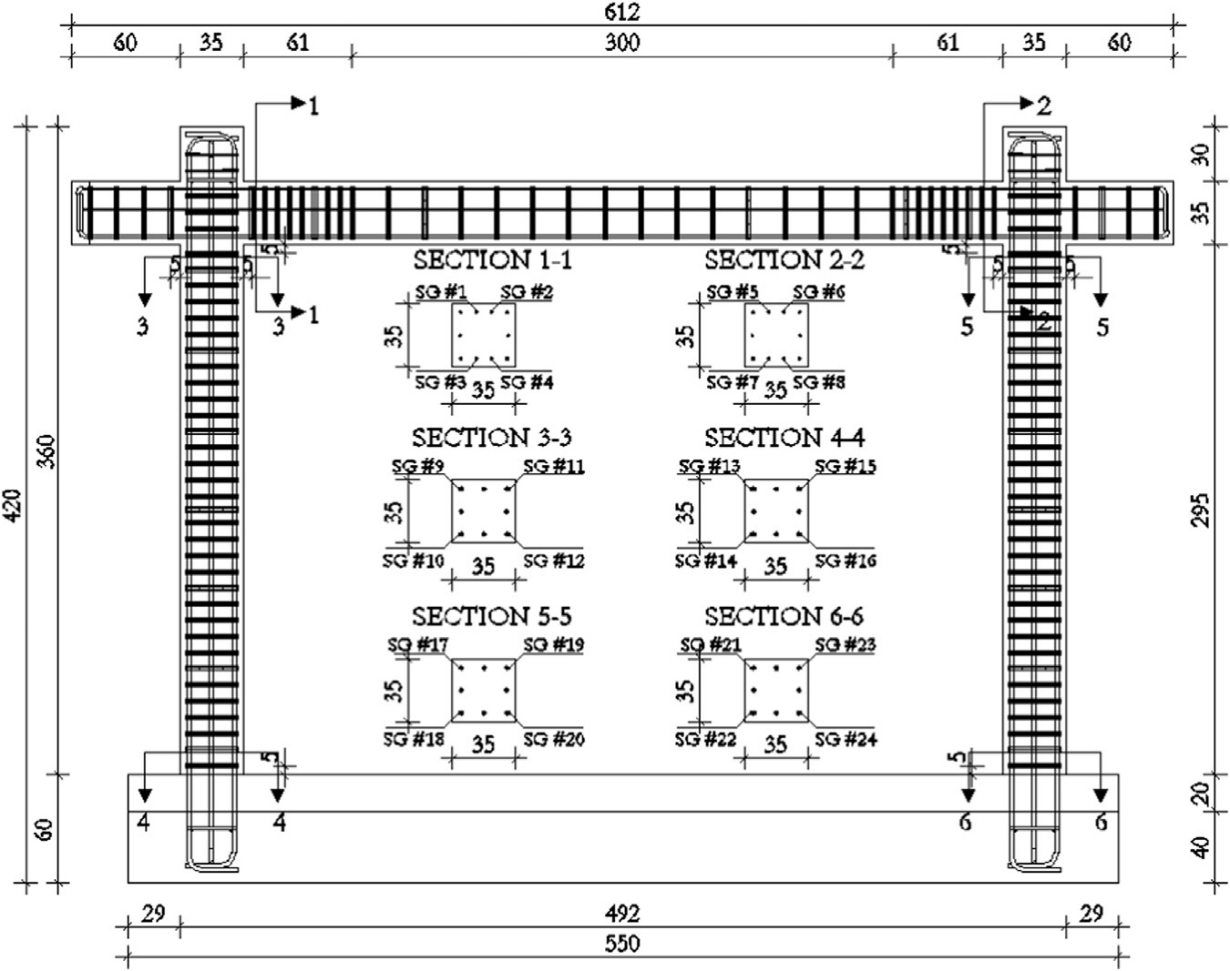
Layout of strain gauges installed on the RC frame reinforcement (TNT, TA1, TA2).

Additionally, during the in-plane test on the bare frame (TNT_IP) and on two infilled frame configurations (TA1_IP, TA2_IP) the deformations of the reinforcement rebars in potential plastic hinge regions of the RC structural elements have been monitored my means of strain gauges attached during the construction of the specimens. In particular, four strain gauges were installed at each of the beam and column ends, resulting in a total of 24 instruments per specimen, as illustrated in Fig. 13.

**Fig. 14:**
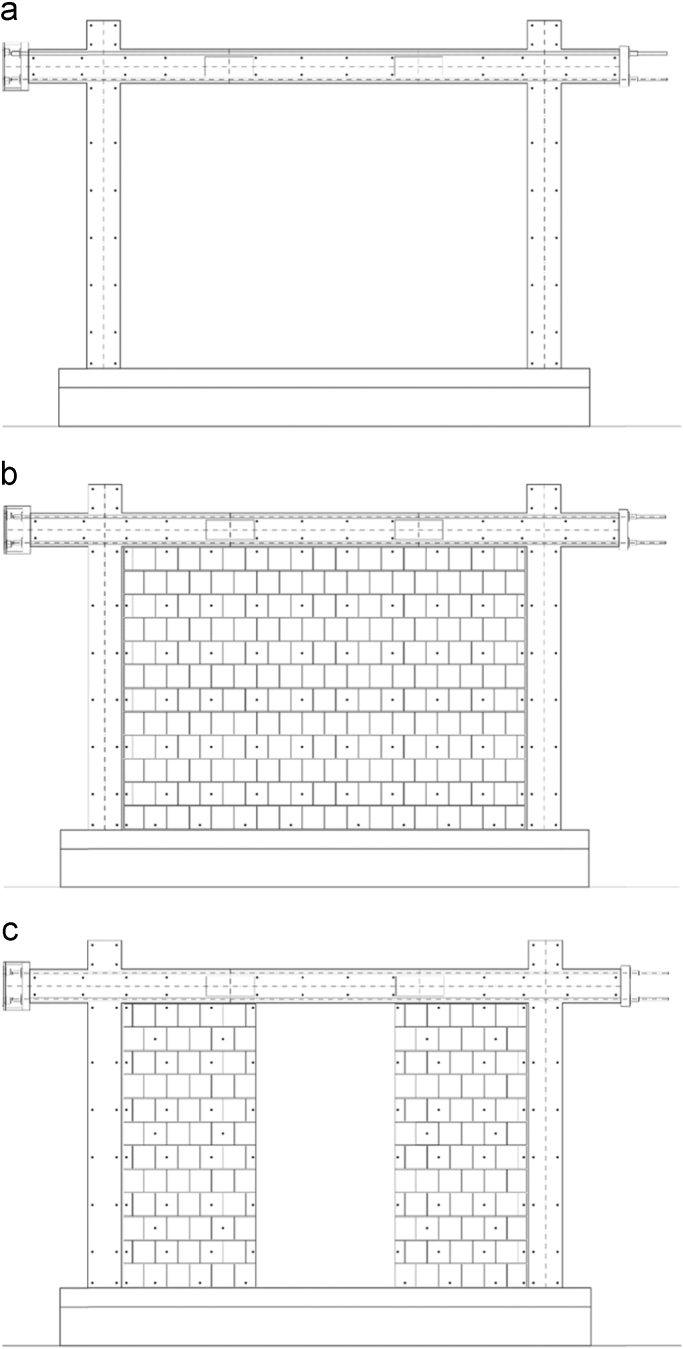
Layout of the optical markers installed on TNT (a), TA2 (b) and TA4 (c).

Finally, an optical acquisition system has been installed to measure the in-plane displacements of optical markers uniformly distributed (about 40 to 50 cm spaced) on the RC frame as well as on the masonry infill, as illustrated in Fig. 14.

#### 2.2.3 Force-displacement response

The results of cyclic in-plane tests on bare and infilled frame configurations are here presented in terms of raw data of hysteretic force-displacement curves. The top displacement of the frame specimen is measured at the centreline of the top beam (potentiometer 13) that corresponds to the centre of the horizontal actuator. The F-D curves processed starting from the following data have been reported in Morandi et al. [1].

##### 2.2.3.1 Bare frame TNT

**Fig. 15:**
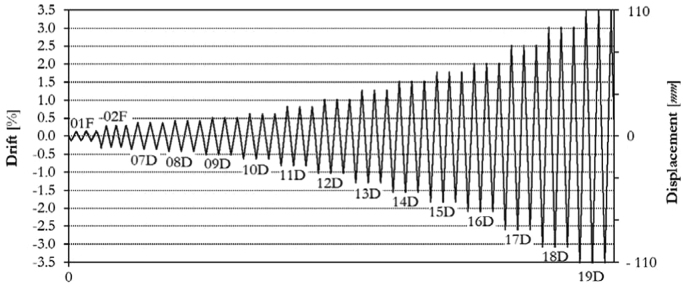
Drift/displacement history – Bare frame TNT.

**Fig. 16:**
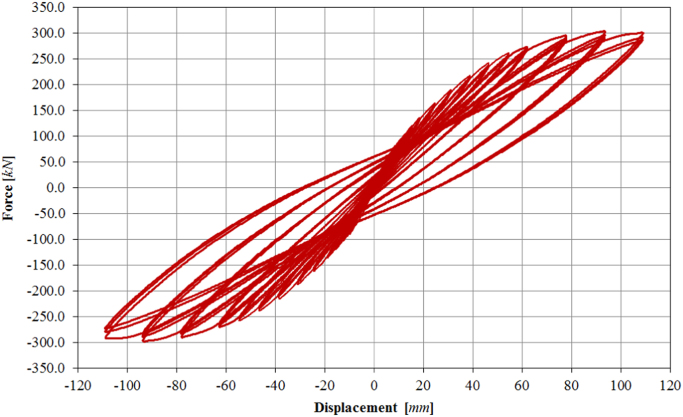
In-plane test results – Bare frame TNT.

Following the given loading history scheme (Table 8), the bare frame (specimen TNT) has been loaded at two different force-controlled loading levels (assuming 0.15*F*_*max*_ ≈ 40.0 kN, 0.30*F*_*max*_ ≈ 80.0 kN) and subsequently at thirteen different target displacements (07D, 08D, 09D, 10D, 11D, 12D, 13D, 14D, 15D, 16D, 17D, 18D, 19D), up to a maximum drift of 3.50%. The complete displacement history is shown in Fig. 15, while the obtained corresponding in-plane force-displacement response is shown in Fig. 16.

##### 2.2.3.2 Infilled frame TA1_IP

**Fig. 17:**
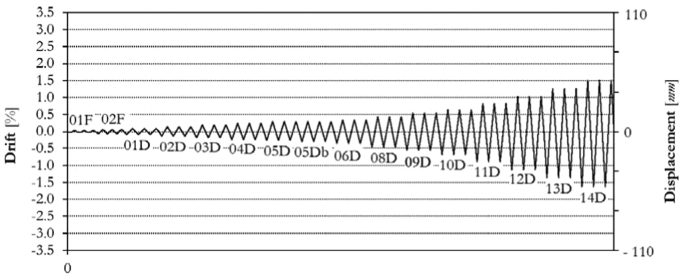
Drift/displacement history – Infilled frame TA1_IP.

**Fig. 18:**
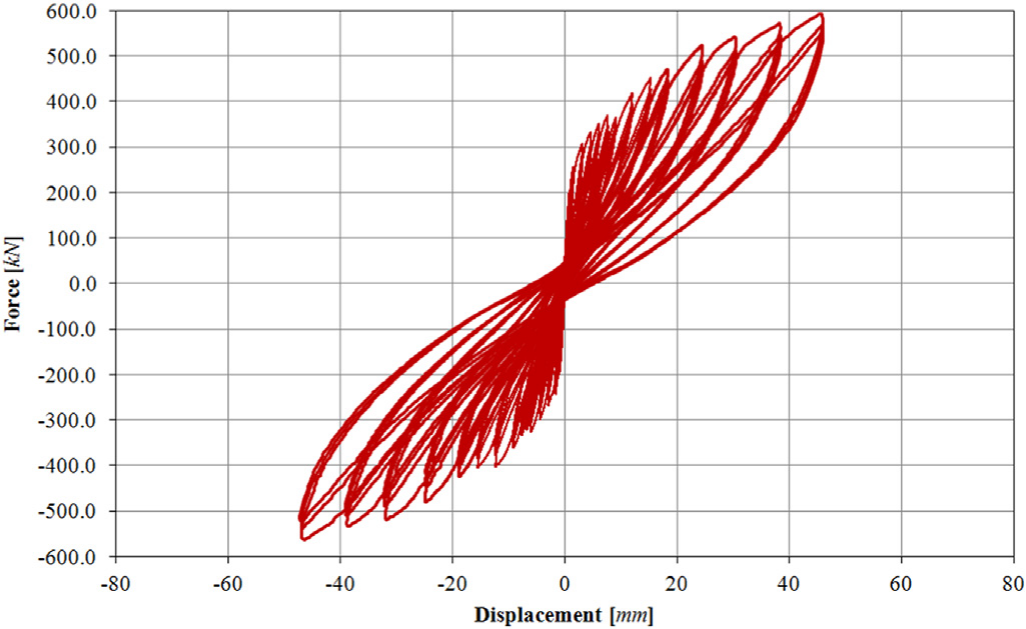
In-plane test results – Infilled frame TA1_IP.

The infilled frame specimen TA1 has been loaded at two different force-controlled loading levels (assuming 0.15*F*_*max*_ ≈ 100.0 kN, 0.30*F*_*max*_ ≈ 200.0 kN) and subsequently at thirteen different target displacements (01D, 02D, 03D, 04D, 05D, 06D, 08D, 09D, 10D, 11D, 12D, 13D, 14D), up to a maximum drift of 1.50%. The complete displacement history is shown in Fig. 17. The obtained corresponding in-plane force-displacement response is shown in Fig. 18.

##### 2.2.3.3 Infilled Frame TA2_IP

**Fig. 19:**
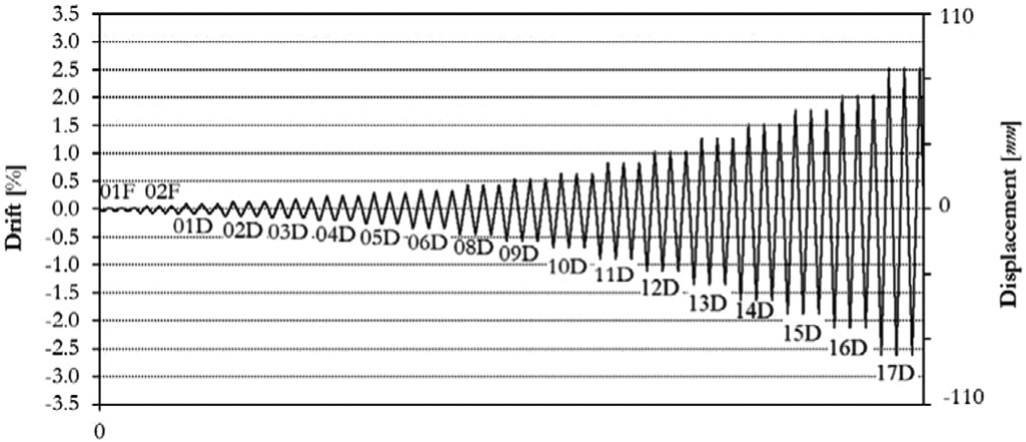
Drift/displacement history – Infilled frame TA2_IP.

**Fig. 20:**
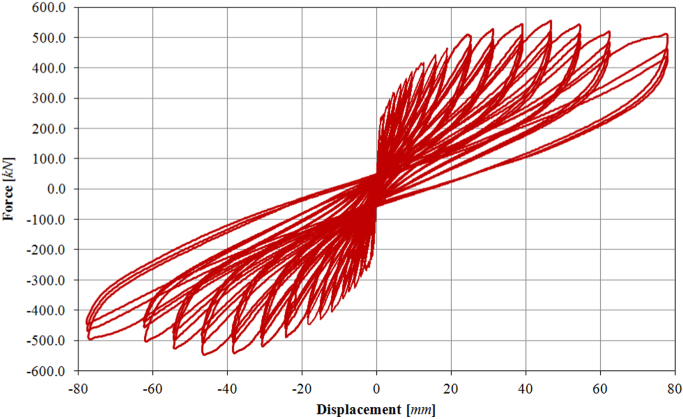
In-plane test results – Infilled frame TA2_IP.

The infilled frame specimen TA2 has been loaded at two different force-controlled loading levels, for 0.15*F*_*max*_ ≈ 100.0 kN and 0.30*F*_*max*_ ≈ 200.0 kN, and afterwards at eighteen different target displacements (01D, 02D, 03D, 04D, 05D, 06D, 08D, 09D, 10D, 11D, 12D, 13D, 14D, 15D, 16D, 17D), up to a maximum drift of 2.50%, as illustrated by the loading history in Fig. 19. The related in-plane force-displacement response is shown in Fig. 20.

##### 2.2.3.4 Infilled frame TA3_IP

**Fig. 21:**
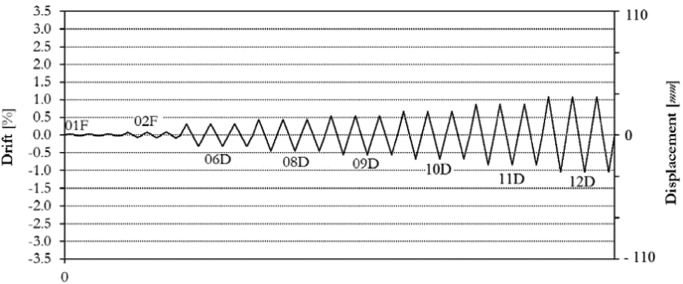
Drift/displacement history – Bare frame TA3_IP.

**Fig. 22:**
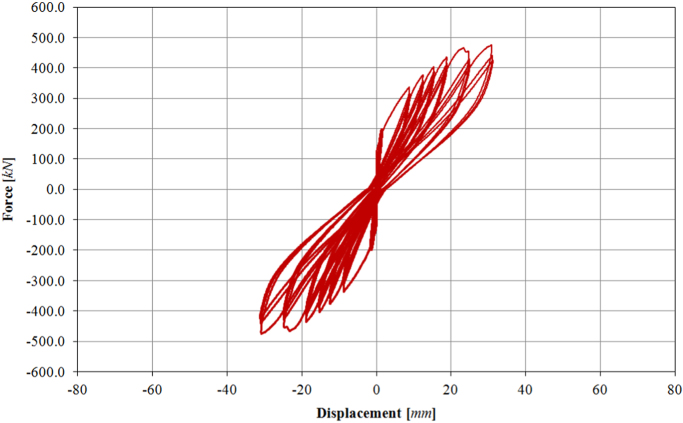
In-plane test results – Infilled frame TA3_IP.

Also for the infilled frame specimen TA3, two levels of force-controlled loading have been accomplished, for 0.15*F*_*max*_ ≈ 100.0 kN and 0.30*F*_*max*_ ≈ 200.0 kN. Subsequently, the specimen has been loaded at six different target displacements (06D, 08D, 09D, 10D, 11D, 12D), up to a maximum drift of 1.00%, as illustrated by the loading history in Fig. 21. The related in-plane force-displacement response is shown in Fig. 22.

##### 2.2.3.5 Infilled frame TA4_IP

**Fig. 23:**
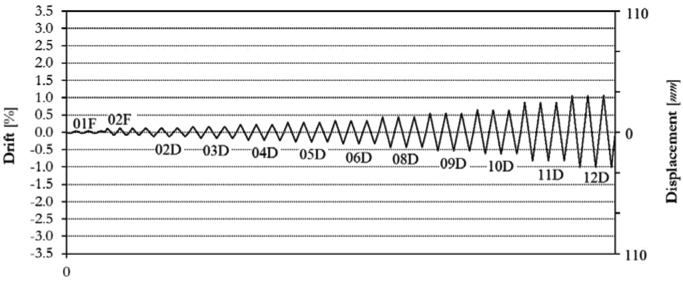
Drift/displacement history – Infilled frame TA4_IP.

**Fig. 24:**
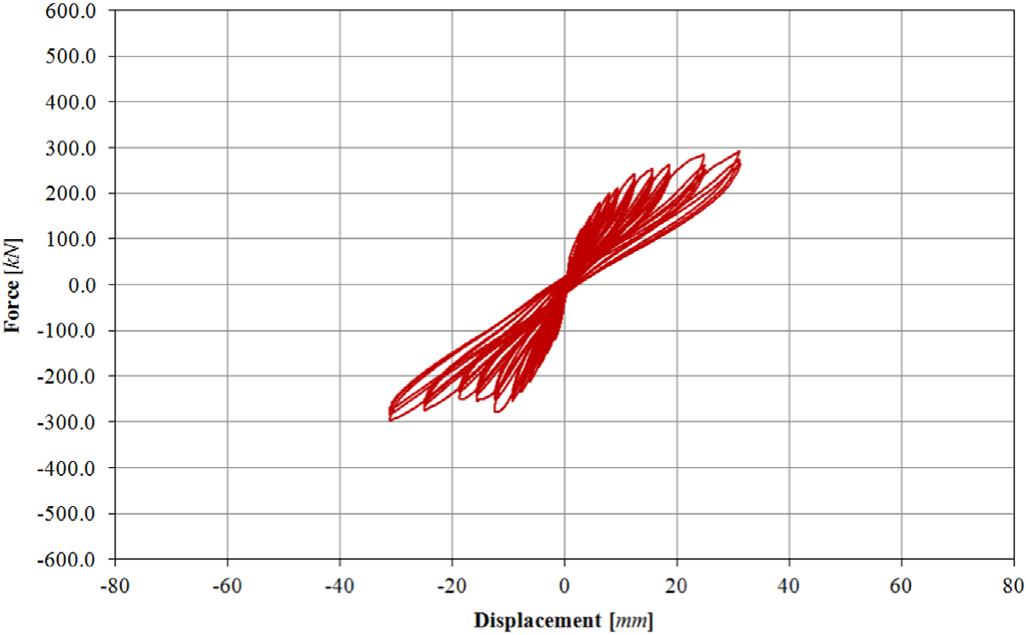
In-plane test results – Infilled frame TA4_IP.

Initially, two levels of force-controlled loading have been carried out, for 0.15*F*_*max*_ ≈ 60.0 kN and 0.30*F*_*max*_ ≈ 120.0 kN. Following the force-controlled loading, the specimen has been loaded at ten different target displacements (02D, 03D, 04D, 05D, 06D, 08D, 09D, 10D, 11D, 12D), up to a maximum drift of 1.00%, as illustrated by the loading history in Fig. 23. The related in-plane force-displacement response is shown in Fig. 24. The first level of force loading has been started in the push direction, while for all subsequent cycles the usual loading direction (first pull, the push) has been adopted.

## Appendix A Supplementary material

Supplementary material In the attached spreadsheet, the data of the force-displacement curves on specimens TNT, TA2 and TA4 have been reported. The horizontal displacement has been measured at the beam centreline, in correspondence of potentiometer 13.
